# Genome-wide Mendelian randomization identifies putatively causal gut microbiota for multiple peptic ulcer diseases

**DOI:** 10.3389/fimmu.2023.1260780

**Published:** 2023-10-05

**Authors:** Jingwei Zhao, Yucheng Hou, Tianyi Xie, Yizhang Zhu, Xinyi Feng, Yong Zhang, Ziyi Yang, Wei Gong

**Affiliations:** ^1^ Department of General Surgery, Xinhua Hospital, Affiliated to Shanghai Jiao Tong University School of Medicine, Shanghai, China; ^2^ Shanghai Key Laboratory of Biliary Tract Disease Research, Shanghai, China; ^3^ Department of Cardiovascular Surgery, The First Affiliated Hospital of Soochow University, Suzhou, Jiangsu, China; ^4^ Laboratory of Medical Science, School of Medicine, Nantong University, Nantong, Jiangsu, China; ^5^ Department of General Surgery, The First Affiliated Hospital of Soochow University, Suzhou, Jiangsu, China; ^6^ Department of Gastroenterology, Wuzhong People’s Hospital of Suzhou, Suzhou, China

**Keywords:** Mendelian randomization, gut microbiota, peptic ulcer, causal relationship, genus

## Abstract

**Objective:**

The pathogenesis of peptic ulcer diseases (PUDs) involves multiple factors, and the contribution of gut microbiota to this process remains unclear. While previous studies have associated gut microbiota with peptic ulcers, the precise nature of the relationship, whether causal or influenced by biases, requires further elucidation.

**Design:**

The largest meta-analysis of genome-wide association studies was conducted by the MiBioGen consortium, which provided the summary statistics of gut microbiota for implementation in the Mendelian randomization (MR) analysis. Summary statistics for five types of PUDs were compiled using the FinnGen Consortium R8 release data. Various statistical techniques, including inverse variance weighting (IVW), MR-Egger, weighted median (WM), weighted mode, and simple mode, were employed to assess the causal relationships between gut microbiota and these five PUDs.

**Result:**

In the intestinal microbiome of 119 known genera, we found a total of 14 causal associations with various locations of PUDs and reported the potential pathogenic bacteria of *Bilophila* et al. Among them, four had causal relationships with esophageal ulcer, one with gastric ulcer, three with gastroduodenal ulcer, four with duodenal ulcer, and two with gastrojejunal ulcer.

**Conclusion:**

In this study, the pathogenic bacterial genera in the gut microbiota that promote the occurrence of PUDs were found to be causally related. There are multiple correlations between intestinal flora and PUDs, overlapping PUDs have overlapping associated genera. The variance in ulcer-related bacterial genera across different locations underscores the potential influence of anatomical locations and physiological functions.

## 1 Introduction

Peptic ulcer diseases (PUDs) represent a prevalent clinical condition characterized by multifactorial etiology and extremely complex pathogenesis, primarily related to *Helicobacter pylori* infection (1). The incidence of PUDs is common among individuals between the age of 25 and 64 years and increases with age. These ulcers are predominantly located near the stomach or duodenum but can also occur in the esophagus or Meckel’s diverticulum (2). In the general population, the lifetime prevalence of PUDs is estimated to range between 5% and 10%, while the annual incidence rate ranges from 0.1% to 0.3% (3). The continued prevalence of peptic ulcers within the stomach and duodenum poses a significant threat to global public health. The diagnosis and treatment of PUDs remain a major healthcare problem with a significant disease burden (2).

The human gut harbors an intricate and diverse microbial community that plays a crucial role in both health and diseases (4, 5), for instance, digestion and absorption of substances, synthesis of essential vitamins such as B and K, catabolism of compounds *in vivo*, coordination of innate and cell-mediated immune responses, and maintenance of intestinal barrier function (6). The symbiotic relationship between these microbes and the host is indispensable for maintaining overall homeostasis; disruptions in this ecological equilibrium can lead to adverse health outcomes (7). The correlation between alterations in the gut microbiome and peptic ulcers has been studied for a long time. Several studies have demonstrated the mechanism underlying *H. pylori*-induced PUDs (8). At the same time, histological techniques have been utilized to examine the microbiome and metabolome of gastric biopsy tissues, identifying a distinct correlation between gastrointestinal ulcers and gastrointestinal bacteria (6). Moreover, PUDs were significantly associated with abnormal microbiota compositions in the oropharynx, esophagus, and gastrointestinal tract (9). Therapies to protect, adapt, shape, or restore the balance of the microbiome are critical aspects of the current and prospective approaches to gastrointestinal ulcer management (10). However, the causal relationship between PUDs at different anatomical sites and the gut microbiota remains unclear and requires further elucidation.

The genome-wide association study (GWAS) has gained widespread acceptance as a pivotal approach for exploring potential genetic variants linked to diverse and complex traits and diseases (11, 12). Mendelian randomization (MR) analysis introduces an innovative paradigm to explore the potential causal association between exposure and outcome independent of confounding factors and ethical considerations. Through MR, genetic variants are leveraged as instrumental variables (IVs) for exposure, enabling the estimation of causality between the exposure and the resultant outcome (13, 14). An MR study mimics a randomized controlled trial (RCT), as genetic variations are randomly assigned during fertilization (15). Furthermore, genotype formation occurs prior to disease onset and is typically unaffected by disease progression, reducing the likelihood of confounding influences.

Here, we employed MR analysis to investigate the correlation between gut microbiota and PUDs. We further explored the potential therapeutic implications of selective support or disorder of the gut microbiota.

## 2 Methods

### 2.1 Study design

**Figure 1:**
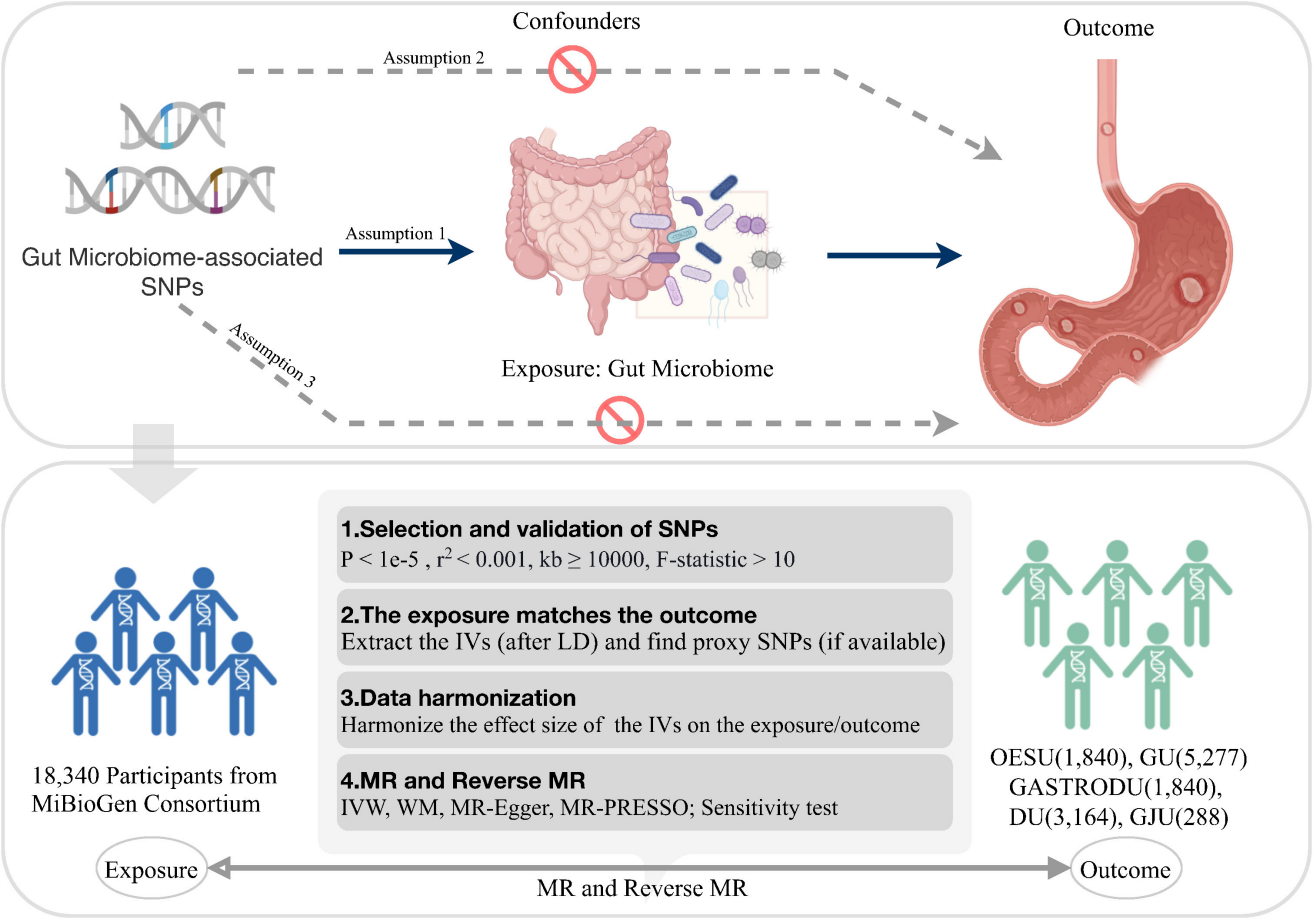
Overview of the study logic and workflow. SNP, single nucleotide polymorphism; LD, linkage disequilibrium; IVW, inverse variance weighted; WM, weighted median; MR-PRESSO, Mendelian Randomization Pleiotropy RESidual Sum and Outlier; MR, Mendelian randomization. Created with BioRender.com.

The basic logic and analysis flow of the entire procedure were briefly described in **Figure 1**. The causal effects of the gut microbiota on five PUDs, including esophageal ulcer (OESU), gastric ulcer (GU), gastroduodenal ulcer (GASTRODU), duodenal ulcer (DU), and gastrojejunal ulcer (GJU), were evaluated. To comprehensively investigate the role of the gut microbiota in PUDs, MR analysis was performed at the classification of genera. The population information involved in the MR was detailed in **Table 1**.

**Table 1:** The population information involved in this Mendelian randomization.

Exposure/Outcome	Ethnic origin	Sample size (case/control)	Gender	Registry filter (ICD-10)	Public release	Data source
Gut microbiota (genus)	European, Hispanic, Middle Eastern, Asian and African	18,340	Mixed sex	—	2018	MiBioGen consortium
OESU	Europe	1,840/292,256	Mixed sex	K22.1	2022	FinnGen R8
GU	Europe	5,277/292,256	Mixed sex	K25	2022	FinnGen R8
GASTRODU	Europe	8,240/292,256	Mixed sex	K2[5-8]	2022	FinnGen R8
DU	Europe	3,164/292,256	Mixed sex	K26	2022	FinnGen R8
GJU	Europe	288/292,256	Mixed sex	K28	2022	FinnGen R8

OESU, esophageal ulcer; GU, gastric ulcer; GASTRODU, gastroduodenal ulcer; DU, duodenal ulcer; GJU, gastrojejunal ulcer; IVW, inverse variance weighted; SNP, single nucleotide polymorphism.

### 2.2 Data sources

A two-sample MR study was undertaken to explore the potential relationship between genus-level gut microbiota and PUDs, utilizing GWAS summary data. Studies received prior approval from their respective institutional review boards (IRBs), and informed consent was obtained from all participants and/or their legal guardians.

To obtain GWAS summary statistics for the gut microbiota, data from the MiBioGen consortium, the largest GWAS dataset published to date, were utilized (16). This dataset consisted of 18,340 individuals spanning 24 population-based cohorts of diverse ancestry, including European, Middle Eastern, East Asian, American Hispanic/Latino, and American African. Microbial composition profiling and taxonomic classification were performed using direct taxonomic binning, targeting variable regions V4, V3–V4, and V1–V2 of the 16S rRNA gene. A microbiota quantitative trait loci (mbQTL) mapping analysis was employed to identify host genetic variants associated with the abundance of bacterial taxa within the gut microbiota. The genus with the least classification in the GWAS data for gut microbiota was selected for preprocessing. Out of 131 identified genera with an average abundance surpassing 1%, 119 genera were included for analysis, while 12 genera remain uncharacterized.

For the GWAS summary statistics of the five peptic ulcer types, data were obtained from the FinnGen consortium R8 release (https://www.finngen.fi/fi). The FinnGen consortium is a large public–private partnership aiming to collect and analyze genomic and health data from 500,000 Finnish biobank participants (12). The dataset available for analysis was up to December 2022. The peptic ulcer types included were OESU (NCase = 1,840, NControl = 292,256), GU (NCase = 5,277, NControl = 292,256), GASTRODU (NCase = 8,240, NControl = 292,256), DU (NCase = 3,164, NControl = 292,256), and GJU (NCase = 288, NControl = 292,256). The classification of OESU, GU, GASTRODU, DU, and GJU adhered strictly to the guidelines outlined by the International Classification of Diseases 10th Revision (ICD-10) code.

### 2.3 Instrumental variable selection

The process of IV selection from the GWAS summary statistics of the gut microbiome adhered to the following criteria: 1) Single-nucleotide polymorphisms (SNPs) associated with each genus at the locus-wide significance threshold (P < 1e–5) were considered as potential IVs (17); 2) Using reference panel data from the 1000 Genomes project European samples, linkage disequilibrium (LD) between SNPs was calculated. Among the SNPs with R^2^ < 0.001 (clumping window size = 10,000 kb), only those with the lowest P-values were retained to minimize biased genetic variation arising from residual LD; 3) The intensity of each IV and exclusion of weak instruments were evaluated by calculating the F-statistic (F > 10); 4) In the presence of palindromic SNPs, the alleles on the forward strand were deduced by utilizing information on allele frequencies; 5) In cases where exposure-associated SNPs were absent in outcome data, suitable proxy SNPs (r^2^ > 0.8) were identified and included in subsequent analyses; and 6) To address confounding, SNPs related to *H. pylori* infection, bile reflux, obesity, alcoholism, smoking, and stress factors were systematically removed during the MR analysis.

### 2.4 Statistical analysis

**Figure 2:**
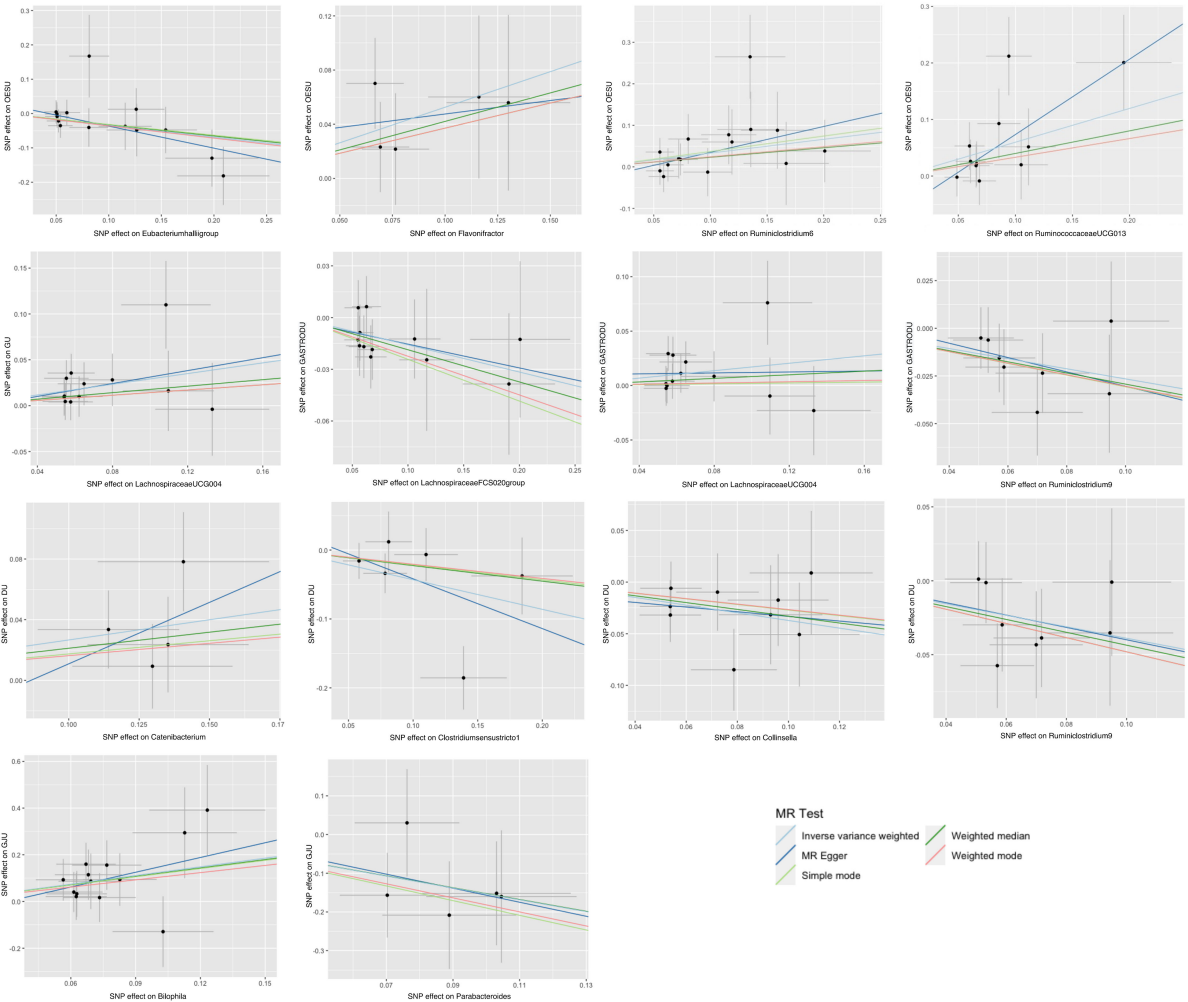
Scatter plots for the causal association between gut microbiota and peptic ulcer.

Five popular MR methods were utilized to analyze valid IVs: inverse variance-weighted (IVW) test, MR-Egger regression, weighted median, weighted mode, and simple mode (**Figure 2**). Among these, IVW was predominantly used due to its slightly higher power under certain conditions (18). The IVW method utilized the inverse of the outcome variance as weights for fitting, regardless of the presence of an intercept term in the regression. Complementary assessments were performed using the remaining four methods, each of which was based on different assumptions about potential pleiotropy. If the results obtained by these complementary methods are consistent with the IVW estimation results, the robustness of the effect estimation can be reinforced.

Multiple methods of sensitivity analyses were undertaken to ensure robustness. Initially, Cochran’s Q statistics was applied to assess heterogeneity across diverse studies (19). Statistically significant Cochran’s Q-test would indicate significant heterogeneity in the analytical outcomes. Secondly, MR-Pleiotropy Residual Sum and Outlier (MR-PRESSO) was used to detect instances of horizontal pleiotropy, with SNPs demonstrating horizontal pleiotropy outliers being systematically excluded to minimize pleiotropy-induced effects (20). In cases where significant horizontal pleiotropy was detected in the MR-PRESSO global test, outliers with P < 0.05 were removed, and the remaining SNPs were reanalyzed with the IVW analysis. Thirdly, the MR-Egger regression intercept was employed to estimate the potential pleiotropy of SNPs, with a P-value >0.05 indicating no horizontal pleiotropy (21). Fourthly, a leave-one-out analysis was performed to assess the impact of each SNP on the causal signal. Finally, funnel and forest plots were constructed to visually examine the presence of horizontal pleiotropy in the MR analysis, with P < 0.05 indicating potential causal associations. The statistical analyses were carried out using the R packages: two-sample MR (22) and MR-PRESSO (20).

## 3 Results

### 3.1 Genetic correlations between gut microbiota and PUDs

Initially, 1,698 SNPs were screened as possible IVs for 131 bacterial genera including 12 unidentified genera. The genetic variants were then eliminated based on specific criteria. All F-statistic exceeded 10, suggesting the absence of weak ins. Following validation through the PhenoScanner database, the remaining SNPs exhibited no discernible associations with *H. pylori* infection, bile reflux, obesity, alcoholism, smoking, and stress factors, indicating that IVs were not resolved by confounding factors. Concurrently, the elimination of palindromic SNPs was performed. GWAS data for patients with PUDs were derived from corresponding cohorts.

### 3.2 Bidirectional causal relationship of gut bacteria on PUD development

Our results demonstrated an association of four bacterial genera with OESU, one with GU, three with GASTRODU, four with DU, and two with GJU. Reverse MR analysis demonstrated that peptic ulcers did not change the abundance of the above bacteria. The leave-one-out sensitivity test highlighted some continuity around the midpoint. Crossing the zero line indicated that the result may be insignificant or unstable. The overall assessment indicated an absence of SNPs having a dominant impact. Furthermore, the selected SNPs exhibited no significant heterogeneity, as indicated by Cochran’s Q statistics. Leave-one-out analysis did not identify a single SNP driving the association (**Supplementary Figure S1**). The application of MR-PRESSO yielded no outliers. The findings from the MR-Egger regression intercept analysis further corroborated the absence of significant directional horizontal pleiotropy (**Table 2**). The three main statistical results of the MR analysis were shown in **Supplementary Table S1**. Given the absence of significant statistical difference in reverse causality, relevant results were presented in **Supplementary Table S2**.

**Table 2:** MR estimates for the association between gut microbiota and PUDs.

Exposure	Outcome	SNP (n)	IVW		Cochran’s Q(MR-Egger)		Pleiotropy_test			F-statistic (median)
			OR (95% CI)	P-value	Q	Q_pval	Egger intercept	Se	P-value	
*Eubacterium hallii*	OESU	14	0.71 (0.53-0.95)	0.024	5.935	0.92	0.028	0.026	0.30	21.10
*Flavonifractor*	OESU	5	1.69 (1.08-2.64)	0.020	1.349	0.72	0.028	0.074	0.73	21.93
*Ruminiclostridium 6*	OESU	15	1.39 (1.03-1.88)	0.030	8.677	0.80	-0.027	0.033	0.42	20.91
*Ruminococcaceae UCG013*	OESU	11	1.82 (1.27-2.61)	0.001	7.770	0.56	-0.060	0.040	0.17	21.52
*Lachnospiraceae UCG004*	GU	12	1.34 (1.09-1.65)	0.006	5.900	0.82	-0.004	0.028	0.89	21.26
*Lachnospiraceae FCS020*	GASTRODU	12	0.85 (0.73-0.99)	0.040	2.979	0.98	-0.002	0.015	0.92	21.66
*Lachnospiraceae UCG004*	GASTRODU	12	1.19 (1.00-1.40)	0.048	8.217	0.61	0.010	0.023	0.68	21.26
*Ruminiclostridium 9*	GASTRODU	8	0.77 (0.61-0.96)	0.019	2.762	0.84	0.007	0.035	0.84	21.46
*Catenibacterium*	DU	4	1.31 (1.05-1.63)	0.018	2.395	0.30	-0.070	0.199	0.76	21.28
*Clostridium sensu stricto 1*	DU	6	0.65 (0.42-1.00)	0.048	9.815	0.04	0.031	0.062	0.65	20.32
*Collinsella*	DU	9	0.69 (0.50-0.95)	0.024	3.549	0.83	-0.011	0.043	0.81	20.78
*Ruminiclostridium 9*	DU	8	0.68 (0.48-0.97)	0.031	3.555	0.74	0.002	0.055	0.97	21.46
*Parabacteroides*	GJU	5	0.22 (0.06-0.84)	0.027	1.590	0.66	0.025	0.374	0.95	21.55
*Bilophila*	GJU	13	3.45 (1.52-7.81)	0.003	7.230	0.78	-0.064	0.150	0.68	21.02

OESU, esophageal ulcer; GU, gastric ulcer; GASTRODU, gastroduodenal ulcer; DU, duodenal ulcer; GJU, gastrojejunal ulcer; IVW, inverse variance weighted; SNP, single nucleotide polymorphism.

#### 3.2.1 Causal relationship of gut bacteria on OESU

Notably, the two-sample MR analysis unveiled a causal linkage between *Eubacterium hallii* and OESU [IVW odds ratio (OR) = 0.71, 95% CI: 0.53–0.95, P = 0.024]. Furthermore, three bacterial features exhibited potential associations with an increased OESU risk: *Flavonifractor* (IVW OR = 1.69, 95% CI: 1.08–2.64, P = 0.020), *Ruminiclostridium 6* (IVW OR = 1.39, 95% CI: 1.03–1.88, P = 0.030), and *Ruminococcaceae UCG013* (IVW OR = 1.82, 95% CI: 1.27–2.61, P = 0.001).

#### 3.2.2 Causal relationship of gut bacteria on GU

Our results underscored a robust causal relationship between *Lachnospiraceae UCG004* and GU (IVW OR = 1.34, 95% CI: 1.09–1.65, P = 0.006).

#### 3.2.3 Causal relationship of gut bacteria on GASTRODU

We found that *Lachnospiraceae UCG004* (IVW OR = 1.19, 95% CI: 1.00–1.40, P = 0.048) was associated with an increased GASTRODU risk. Moreover, *Lachnospiraceae FCS020* (IVW OR = 0.85, 95% CI: 0.73–0.99, P = 0.040) and *Ruminiclostridium 9* (IVW OR = 0.77, 95% CI: 0.61–0.96, P = 0.019) were associated with a lower GASTRODU risk.

#### 3.2.4 Causal relationship of gut bacteria on DU

We found potential associations between one bacterial feature, *Catenibacterium* (IVW OR = 1.31, 95% CI: 1.05–1.63, P = 0.018), and increased DU risk. Meanwhile, three bacterial features, *Clostridium sensu stricto 1* (IVW OR = 0.65, 95% CI: 0.42–1.00, P = 0.048), *Collinsella* (IVW OR = 0.69, 95% CI: 0.50–0.95, P = 0.024), and *Ruminiclostridium 9* (IVW OR = 0.68, 95% CI: 0.48–0.97, P = 0.031), were associated with a reduced DU risk.

#### 3.2.5 Causal relationship of gut bacteria on GJU

Furthermore, we found an association between *Parabacteroides* (IVW OR = 0.22, 95% CI: 0.06–0.84, P = 0.027) and a lower GJU risk, while *Bilophila* (IVW OR = 3.45, 95% CI: 1.52–7.81, P = 0.003) showed a correlation with an elevated risk of GJU.

## 4 Discussion

Prior to the recognition of *H. pylori* infection and the extensive utilization of nonsteroidal anti-inflammatory drugs (NSAIDs) during the latter time frame of the 20th century, PUDs were primarily attributed to a hypersecretory acidic environment, coupled with dietary factors or stress (23, 24). However, there is growing recognition that the etiology of PUDs extends beyond *H. pylori* infection in the stomach (6). Rather, the genesis and progression of PUDs emerge as a result of the interplay of multiple factors, encompassing the presence of different *H. pylori* virulence proteins, ensuing human immune reactions, and imbalances in the gastrointestinal microbiota (1, 25). The role of intrinsic gut bacteria in PUD development is also noteworthy. Our findings emphasized the causal involvement of specific bacterial characteristics’ abundance in modulating the susceptibility to diverse peptic ulcer types. Remarkably, this study represents the first MR analysis to illuminate the multiple connections between gut microbiota and PUDs. We regarded it as a longitudinal microbiome investigation conducted before the onset of PUDs in humans. This study effectively identified robustly gene variants through the largest gut microbiome GWAS.

One pivotal role played by gut microbiota involves the synthesis of short-chain fatty acids (SCFAs), which can directly regulate host health through energy regulation, intestinal mucosal barrier, immune regulation, and induction of tumor cell differentiation and apoptosis (26, 27). Dysregulation in the equilibrium of SCFAs within the body results in a cascade of disease manifestations (28). Moreover, SCFAs can promote the expression of tight junction proteins, such as claudin, occludin, and Zonula occludens (ZOs) within the intestinal tract, decrease intestinal permeability, promote the proliferation of intestinal mucosal cells, and improve the mechanical barrier function of the intestine in animal models (29, 30). In this MR study, SCFA-producing bacteria included *E. hallii* (31), *Flavonifractor* (32), *Ruminiclostridium* (33), *Ruminococcaceae* (33), *Collinsella* (34), and *Parabacteroides* (35).

A previous study revealed reduced levels of *Collinsella* in patients with inflammatory bowel disease (IBD) and gut microbiota dysbiosis (36). This finding was consistent with our results, implicating a potentially beneficial role for *Collinsella* in gut health and highlighting its association with a reduced DU risk. Researchers have found that *Parabacteroides* could produce a molecule named rhamnose in the mouse gut to facilitate the repair and maintenance of the intestinal mucosal barrier in mice (37). This suggested that *Parabacteroides* may benefit gut health, aligning with our findings that it exhibited a negative association with GJU. *E. hallii* is a high-yielding butyrate producer in the gut, contributing significantly to the maintenance of intestinal metabolic equilibrium (38). In the context of aging populations characterized by a decrease in microbiota diversity, a reduction in the abundance of *E. hallii* has been noted, accompanied by decreased production of SCFAs and increased intestinal inflammation (39). Consistent with these studies, our results demonstrated a negative link with OESU. The presence of *Eubacterium* in the gut is primarily associated with increased dietary fiber intake. As previously reported, it may improve the intestinal mucosal barrier and metabolic diseases, making it a potential candidate strain for a new generation of probiotics (40).

Previous studies have shown an increased level of *Flavonifractor* in patients with early-onset colon cancer, while *Lachnospiraceae UCG004* was significantly increased in patients with postmenopausal osteoporosis (PMO). While the adverse effects of *Flavonifractor* remain relatively underexplored, several studies hint at its role in stabilizing gut intestinal flora and immune modulation (41). Our study uncovered a positive correlation between *Flavonifractor* and OESU, hinting at its potential as a risk factor. *Ruminiclostridium*, a common anaerobic intestinal bacterium, plays a pivotal role in polysaccharide degradation and SCFA production, thereby influencing intestinal peristalsis, intestinal health, and immune modulation (42). In addition, *Ruminiclostridium 9* inhibits the growth of other harmful bacteria, which is crucial for preserving gut microbiota equilibrium (43). Recent studies have shown that reduced *Ruminiclostridium 9* abundance is also associated with some intestinal diseases, such as IBD and obesity (10). Our MR study demonstrated it with the negative causal relationship between GASTRODU or DU, thus highlighting the potential protective role of *Ruminiclostridium 9* during the development of PUDs. Despite the positive correlation between *Ruminiclostrium 6* and OESU, we did not observe any significant negative effect in the gut. *Ruminococcaceae* and *Lachnospiraceae* are typical intestinal flora that are important in maintaining intestinal health (44). However, some studies have shown the increased abundance of *Ruminococcaceae* or *Lachnospiraceae* in metabolic disorders such as obesity and diabetes (45). Additionally, certain members of these genera have been associated with the production of inflammatory mediators, enterotoxins, and other harmful substances related to the occurrence and development of intestinal diseases (46, 47). Our study found that *Ruminococcaceae UCG013* was positively associated with OESU, while *Lachnospiraceae UCG004* was positively correlated with GU and GASTRODU, suggesting their potential role as risk factors. Intriguingly, *Lachnospiraceae FCS020* exhibited a negative causal link with OESU.

*C. sensu stricto*, a beneficial intestinal bacterium, has many vital physiological and metabolic functions, such as participating in the metabolism of glucose and lactose as well as promoting the synthesis of biotin and vitamin K (48). *C. sensu stricto* can also promote the integrity of the intestinal mucosal barrier, regulate the intestinal immune response, and reduce intestinal inflammation (48). Our MR analysis corroborates its protective effect, revealing a negative correlation between *C. sensu stricto 1* and DU. Conversely, *Catenibacterium* shows a positive causal association with DU. *Catenibacterium* is a Gram-positive bacterium (49). Although many species of *Catenibacterium* are unknown, different genera play different roles in intestinal diseases. For instance, certain strains have been implicated in the occurrence and development of IBD, wherein harmful substances, such as enterotoxins, contribute to intestinal mucosal barrier disruption and aggravated inflammatory responses (50). Our findings implicate *Bilophila* as a potential risk factor for GJU, which used to be mainly associated with metabolic diseases (51). *Bilophila*’s role in the gut requires further understanding; however, studies suggest that some members of *Bilophila* may be involved in the occurrence and development of intestinal inflammation. Some of these strains can produce harmful substances, such as hydrogen sulfide, breaking the intestinal mucosal barrier and increasing inflammatory responses (52). Notably, PUDs exhibited no significant association with the aforementioned bacteria in reverse MR analysis.

The variance in ulcer-related bacterial genera across different locations underscores the potential influence of anatomical locations and physiological functions. Interestingly, ulcers with overlapping sites, such as gastroduodenal ulcer, gastric ulcer, and duodenal ulcer, exhibited similar bacterial flora and associations, confirming the reliability of the results. Importantly, these findings prompted an exploration into the pathological mechanism underlying overlapping bacterial flora in ulcer development. Furthermore, there was no evidence of reverse causality between PUDs and gut bacterial genera. Overall, gene-based analysis from 119 gut bacterial genera revealed specific genera associated with PUDs in different locations and explained the multiple correlations between them. These findings supported the influence of gut microbiota on the development of PUDs and highlighted the putative association between specific bacterial genera and site-specific PUDs. Ultimately, these findings extended valuable implications for the clinical management of patients afflicted with PUDs.

## 5 Article summary

This study presented a comprehensive analysis of the causal relationships between 121 known gut bacterial genera and PUDs, utilizing both forward and reverse MR analyses. This approach effectively mitigated the influence of confounding variables and causal inference’s challenge of reverse causation. Notably, the genetic variants associated with gut microbiota were derived from the most extensive GWAS meta-analysis, enhancing robust IVs for the MR analysis. Multiple statistical methods were used to test the sensitivity, pleiotropy, and heterogeneity of this study.

However, despite our efforts to minimize confounding influences, the complete elimination of horizontal pleiotropy remains a challenge, largely attributed to our limited understanding of the disease. As knowledge and awareness evolve over time, perceptions about confounding factors may change. In the future, extending MR investigations on the causal relationship between gut microbiota and peptic ulcers in diverse European and non-European populations would enhance the generalization of our findings. Additionally, while this article only explores the problem from the perspective of genetics, higher-level RCTs are necessary to validate the causal relationship while also into the intricate mechanisms underlying specific bacterial contributions.

In summation, through a systematic investigation, this study constituted a pioneering MR analysis focused on gut microbiota and PUDs. Our findings shed light on the multiple correlations between gut microbiota genera and five PUD types. Moreover, it has established definitive links between 14 specific bacterial genera and their corresponding ulcer manifestations, the pathogenic intestinal bacteria deserved more attention. These findings hold significant implications for understanding the role of gut flora in PUDs, offering valuable insights for the formulation of preventive strategies in patients with this condition. Novel treatment avenues targeting specific intestinal bacterial genera may represent new treatment options for PUDs.

## Data availability statement

Publicly available datasets were analyzed in this study. This data can be found here: MiBioGen Consortium, https://mibiogen.gcc.rug.nl/ and FinnGen Consortium R8, https://r8.finngen.fi/.

## Ethics statement

All studies were previously approved by respective Institutional Review Boards (IRBs). No new IRB approval was required. The studies were conducted in accordance with the local legislation and institutional requirements. Written informed consent for participation was not required from the participants or the participants’ legal guardians/next of kin in accordance with the national legislation and institutional requirements.

## Author contributions

JZ: Conceptualization, Data curation, Formal Analysis, Methodology, Software, Validation, Writing – original draft, Writing – review & editing. YH: Data curation, Software, Writing – original draft, Writing – review & editing. YiZ: Conceptualization, Writing – original draft, Writing – review & editing. TX: Conceptualization, Data curation, Software, Writing – original draft. XF: Data curation, Formal Analysis, Software, Writing – original draft. ZY: Conceptualization, Investigation, Supervision, Visualization, Writing – original draft. YaZ: Funding acquisition, Investigation, Resources, Software, Supervision, Writing – review & editing. WG: Funding acquisition, Resources, Supervision, Writing – review & editing.

## Supplementary material

Supplementary Figure 1 Sensitivity test performed by leave-one-out analysis.

## References

[1] Lanas A Chan FKL. Peptic ulcer disease. Lancet (2017) 390(10094):613–24. doi: 10.1016/S0140-6736(16)32404-7 28242110

[2] Ren J Jin X Li J Li R Gao Y Zhang J. The global burden of peptic ulcer disease in 204 countries and territories from 1990 to 2019: a systematic analysis for the Global Burden of Disease Study 2019. Int J Epidemiol (2022) 51(5):1666–76. doi: 10.1093/ije/dyac033 35234893

[3] Lau JY Sung J Hill C Henderson C Howden CW Metz DC. Systematic review of the epidemiology of complicated peptic ulcer disease: incidence, recurrence, risk factors and mortality. Digestion (2011) 84(2):102–13. doi: 10.1159/000323958 21494041

[4] Lozupone CA Stombaugh JI Gordon JI Jansson JK Knight R. Diversity, stability and resilience of the human gut microbiota. Nature (2012) 489(7415):220–30. doi: 10.1038/nature11550 PMC3577372 22972295

[5] Round JL Mazmanian SK. The gut microbiota shapes intestinal immune responses during health and disease. Nat Rev Immunol (2009) 9(5):313–23. doi: 10.1038/nri2515 PMC4095778 19343057

[6] Wang C Yu X Lin HQ Wang GQ Liu JM Gao CC. Integrating microbiome and metabolome revealed microbe-metabolism interactions in the stomach of patients with different severity of peptic ulcer disease. Front Immunol (2023) 14. doi: 10.3389/fimmu.2023.1134369 PMC10034094 36969184

[7] Huang Y Wang Z Ma H Ji S Chen Z Cui Z. Dysbiosis and implication of the gut microbiota in diabetic retinopathy. Front Cell Infect Microbiol (2021) 11. doi: 10.3389/fcimb.2021.646348 PMC8017229 33816351

[8] NIH Consensus Conference. Helicobacter pylori in peptic ulcer disease. NIH consensus development panel on helicobacter pylori in peptic ulcer disease. Jama (1994) 272(1):65–9. doi: 10.1001/jama.1994.03520010077036 8007082

[9] Graham DY. Campylobacter pylori and peptic ulcer disease. Gastroenterology (1989) 96(2 Pt 2 Suppl):615–25. doi: 10.1016/S0016-5085(89)80057-5 2642447

[10] Belizario JE Napolitano M. Human microbiomes and their roles in dysbiosis, common diseases, and novel therapeutic approaches. Front Microbiol (2015) 6. doi: 10.3389/fmicb.2015.01050 PMC4594012 26500616

[11] Howie BN Donnelly P Marchini J. A flexible and accurate genotype imputation method for the next generation of genome-wide association studies. PloS Genet (2009) 5(6):e1000529. doi: 10.1371/journal.pgen.1000529 19543373 PMC2689936

[12] Kurki MI Karjalainen J Palta P Sipila TP Kristiansson K Donner KM. FinnGen provides genetic insights from a well-phenotyped isolated population. Nature (2023) 613(7944):508. doi: 10.1038/s41586-022-05473-8 36653562 PMC9849126

[13] Emdin CA Khera AV Kathiresan S. Mendelian randomization. Jama J Am Med Assoc (2017) 318(19):1925–6. doi: 10.1001/jama.2017.17219 29164242

[14] Gao K Cao LF Ma WZ Gao YJ Luo MS Zhu J. Association between sarcopenia and cardiovascular disease among middle-aged and older adults: Findings from the China health and retirement longitudinal study. Eclinicalmedicine (2022) 44:101264. doi: 10.1016/j.eclinm.2021.101264 35059617 PMC8760427

[15] Greenland S. An introduction to instrumental variables for epidemiologists. Int J Epidemiol (2000) 29(4):722–9. doi: 10.1093/ije/29.4.722 10922351

[16] Kurilshikov A Medina-Gomez C Bacigalupe R Radjabzadeh D Wang J Demirkan A. Large-scale association analyses identify host factors influencing human gut microbiome composition. Nat Genet (2021) 53(2):156–65. doi: 10.1038/s41588-020-00763-1 PMC8515199 33462485

[17] Sanna S van Zuydam NR Mahajan A Kurilshikov A Vila AV Vosa U. Causal relationships among the gut microbiome, short-chain fatty acids and metabolic diseases. Nat Genet (2019) 51(4):600. doi: 10.1038/s41588-019-0350-x 30778224 PMC6441384

[18] Boehm FJ Zhou X. Statistical methods for Mendelian randomization in genome-wide association studies: A review. Comput Struct Biotechnol J (2022) 20:2338–51. doi: 10.1016/j.csbj.2022.05.015 PMC9123217 35615025

[19] Del Greco MF Minelli C Sheehanc NA Thompsonc JR. Detecting pleiotropy in Mendelian randomisation studies with summary data and a continuous outcome. Stat Med (2015) 34(21):2926–40. doi: 10.1002/sim.6522 25950993

[20] Verbanck M Chen C-Y Neale B Do R. Detection of widespread horizontal pleiotropy in causal relationships inferred from Mendelian randomization between complex traits and diseases. Nat Genet (2018) 50(5):693. doi: 10.1038/s41588-018-0099-7 29686387 PMC6083837

[21] Burgess S Thompson SG. Interpreting findings from Mendelian randomization using the MR-Egger method. Eur J Epidemiol (2017) 32(5):377–89. doi: 10.1007/s10654-017-0255-x PMC5506233 28527048

[22] Hemani G Zhengn J Elsworth B Wade KH Haberland V Baird D. The MR-Base platform supports systematic causal inference across the human phenome. Elife (2018) 7:e34408. doi: 10.7554/eLife.34408 29846171 PMC5976434

[23] Suerbaum S Michetti P. Helicobacter pylori infection. N Engl J Med (2002) 347(15):1175–86. doi: 10.1056/NEJMra020542 12374879

[24] Peterson WL. Helicobacter pylori and peptic ulcer disease. N Engl J Med (1991) 324(15):1043–8. 10.1056/NEJM199104113241507 2005942

[25] Hu W Deng C Ma ZQ Wang DJ Fan CX Li T. Utilizing melatonin to combat bacterial infections and septic injury. Br J Pharmacol (2017) 174(9):754–68. doi: 10.1111/bph.13751 PMC5387000 28213968

[26] Smith PM Howitt MR Panikov N Michaud M Gallini CA Bohlooly-Y M. The microbial metabolites, short-chain fatty acids, regulate colonic T-reg cell homeostasis. Science (2013) 341(6145):569–73. doi: 10.1126/science.1241165 PMC3807819 23828891

[27] Morrison DJ Preston T. Formation of short chain fatty acids by the gut microbiota and their impact on human metabolism. Gut Microbes (2016) 7(3):189–200. doi: 10.1080/19490976.2015.1134082 26963409 PMC4939913

[28] Tan C Wu Q Wang H Gao X Xu R Cui Z. Dysbiosis of gut microbiota and short-chain fatty acids in acute ischemic stroke and the subsequent risk for poor functional outcomes. J Parenteral Enteral Nutr (2021) 45(3):518–29. doi: 10.1002/jpen.1861 PMC8048557 32473086

[29] den Besten G van Eunen K Groen AK Venema K Reijngoud D-J Bakker BM. The role of short-chain fatty acids in the interplay between diet, gut microbiota, and host energy metabolism. J Lipid Res (2013) 54(9):2325–40. doi: 10.1194/jlr.R036012 PMC3735932 23821742

[30] Hamer HM Jonkers DMAE Bast A Vanhoutvin SALW Fischer MAJG Kodde A. Butyrate modulates oxidative stress in the colonic mucosa of healthy humans. Clin Nutr (2009) 28(1):88–93. doi: 10.1016/j.clnu.2008.11.002 19108937

[31] Engels C Ruscheweyh H-J Beerenwinkel N Lacroix C Schwab C. The Common Gut Microbe Eubacterium hallii also Contributes to Intestinal Propionate Formation. Front Microbiol (2016) 7. doi: 10.3389/fmicb.2016.00713 PMC4871866 27242734

[32] Roses C Cuevas-Sierra A Quintana S Riezu-Boj JI Martinez JA Milagro FI. Gut microbiota bacterial species associated with mediterranean diet-related food groups in a Northern Spanish population. Nutrients (2021) 13(2):636. doi: 10.3390/nu13020636 33669303 PMC7920039

[33] Liu D Li T Zheng H Yin X Chen M Liao Z. Study on alterations of physiological functions in aged constipation rats with fluid-deficiency based on metabonomic and microbiology analysis. Rsc Adv (2017) 7(76):48136–50. doi: 10.1039/C7RA07651G

[34] Chang Y Chen Y Zhou Q Wang C Chen L Di W. Short-chain fatty acids accompanying changes in the gut microbiome contribute to he development o hypertension in patients with preeclampsia. Clin Sci (2020) 134(2):289–302. doi: 10.1042/CS20191253 31961431

[35] Wang K Liao M Zhou N Bao L Ma K Zheng Z. Parabacteroides distasonis Alleviates Obesity and Metabolic Dysfunctions *via* Production of Succinate and Secondary Bile Acids. Cell Rep (2019) 26(1):222. doi: 10.1016/j.celrep.2018.12.028 30605678

[36] Gevers D Kugathasan S Denson LA Vazquez-Baeza Y Van Treuren W Ren BY. The treatment-naive microbiome in new-onset Crohn's disease. Cell Host Microbe (2014) 15(3):382–92. doi: 10.1016/j.chom.2014.02.005 PMC4059512 24629344

[37] Sonnenburg JL Xu J Leip DD Chen CH Westover BP Weatherford J. Glycan foraging in *vivo* by an intestine-adapted bacterial symbiont. Science (2005) 307(5717):1955–9. doi: 10.1126/science.1109051 15790854

[38] Russell WR Hoyles L Flint HJ Dumas M-E. Colonic bacterial metabolites and human health. Curr Opin Microbiol (2013) 16(3):246–54. doi: 10.1016/j.mib.2013.07.002 23880135

[39] Li Y Ma ZQ Jiang SA Hu W Li T Di SY. A global perspective on FOXO1 in lipid metabolism and lipid-related diseases. Prog Lipid Res (2017) 66:42–9. doi: 10.1016/j.plipres.2017.04.002 28392404

[40] Moco S Martin FPJ Rezzi S. Metabolomics view on gut microbiome modulation by polyphenol-rich foods. J Proteome Res (2012) 11(10):4781–90. doi: 10.1021/pr300581s 22905879

[41] Sanchez B. Bile acid-microbiota crosstalk in gastrointestinal inflammation and carcinogenesis: a role for bifidobacteria and lactobacilli? Nat Rev Gastroenterol Hepatol (2018) 15(4):205. doi: 10.1038/nrgastro.2018.23 29512648

[42] Flint HJ Scott KP Duncan SH Louis P Forano E. Microbial degradation of complex carbohydrates in the gut. Gut Microbes (2012) 3(4):289–306. doi: 10.4161/gmic.19897 22572875 PMC3463488

[43] Gutierrez-Calabres E Ortega-Hernandez A Modrego J Gomez-Gordo R Caro-Vadillo A Rodriguez-Bobada C. Gut microbiota profile identifies transition from compensated cardiac hypertrophy to heart failure in hypertensive rats. Hypertension (2020) 76(5):1545–54. doi: 10.1161/HYPERTENSIONAHA.120.15123 32921194

[44] Louis P Young P Holtrop G Flint HJ. Diversity of human colonic butyrate-producing bacteria revealed by analysis of the butyryl-CoA:acetate CoA-transferase gene. Environ Microbiol (2010) 12(2):304–14. doi: 10.1111/j.1462-2920.2009.02066.x 19807780

[45] Qin JJ Li YR Cai ZM Li SH Zhu JF Zhang F. A metagenome-wide association study of gut microbiota in type 2 diabetes. Nature (2012) 490(7418):55–60. doi: 10.1038/nature11450 23023125

[46] Bajaj JS Ridlon JM Hylemon PB Thacker LR Heuman DM Smith S. Linkage of gut microbiome with cognition in hepatic encephalopathy. Am J Physiolol Gastrointest Liver Physiol (2012) 302(1):G168–G75. doi: 10.1152/ajpgi.00190.2011 PMC3345956 21940902

[47] Lepage P Haesler R Spehlmann ME Rehman A Zvirbliene A Begun A. Twin study indicates loss of interaction between microbiota and mucosa of patients with ulcerative colitis. Gastroenterology (2011) 141(1):227–36. doi: 10.1053/j.gastro.2011.04.011 21621540

[48] Zhao F Feng J Li J Zhao L Liu Y Chen H. Alterations of the gut microbiota in Hashimoto's thyroiditis patients. Thyroid (2018) 28(2):175–86. doi: 10.1089/thy.2017.0395 29320965

[49] Kageyama A Benno Y. Catenibacterium mitsuokai gen. nov., sp, nov., a Gram-positive anaerobic bacterium isolated from human faeces. Int J Syst Evol Microbiol (2000) 50:1595–9. doi: 10.1099/00207713-50-4-1595 10939666

[50] Al-Amrah H Saadah O Mosli M Annese V Al-Hindi R Edris S. Composition of the gut microbiota in patients with inflammatory bowel disease in Saudi Arabia: A pilot study. Saudi J Gastroenterol (2023) 29(2):102–10. doi: 10.4103/sjg.sjg_368_22 PMC10270479 36695274

[51] Natividad JM Lamas B Hang Phuong P Michel M-L Rainteau D Bridonneau C. Bilophila wadsworthia aggravates high fat diet induced metabolic dysfunctions in mice. Nat Commun (2018) 9(1):2802. doi: 10.1038/s41467-018-05249-7 30022049 PMC6052103

[52] Feng Z Long W Hao B Ding D Ma X Zhao L. A human stool-derived Bilophila wadsworthia strain caused systemic inflammation in specific-pathogen-free mice. Gut Pathog (2017) 9:59. doi: 10.1186/s13099-017-0208-7 29090023 PMC5657053

