# Supplementary material for: Genome-wide Mendelian randomization identifies putatively causal gut microbiota for multiple peptic ulcer diseases
Source: Front Immunol. 2023 Oct 5;14:1260780. doi: 10.3389/fimmu.2023.1260780 (PMC10586326; doi:10.3389/fimmu.2023.1260780)
Supplement: Supplementary file 2 [file Table_1.pdf]

Stable1: Statistical supplement for the association between gut microbiota and PUDs

| Exposure | Outcome | SNP (n) | MR Egger | | Weighted median | | IVW | |
|---|---|---|---|---|---|---|---|---|
| | | | OR (95% CI) | P-value | OR (95% CI) | P-value | OR (95% CI) | P-value |
| Eubacterium hallii | OESU | 14 | 0.53 (0.28-0.98) | 0.283 | 0.72 (0.49-1.06) | 0.098 | 0.71 (0.53-0.95) | 0.024 |
| Flavonifractor | OESU | 5 | 1.22 (0.21-7.19) | 0.840 | 1.52 (0.89-2.61) | 0.125 | 1.69 (1.08-2.64) | 0.020 |
| Ruminiclostridium 6 | OESU | 15 | 1.87 (0.89-3.92) | 0.123 | 1.26 (0.85-1.87) | 0.251 | 1.39 (1.03-1.88) | 0.030 |
| Ruminococcaceae UCG013 | OESU | 11 | 3.79 (1.35-10.67) | 0.032 | 1.49 (0.91-2.44) | 0.115 | 1.82 (1.27-2.61) | 0.001 |
| Lachnospiraceae UCG004 | GU | 12 | 1.42 (0.60-3.38) | 0.444 | 1.19 (0.91-1.57) | 0.202 | 1.34 (1.09-1.65) | 0.006 |
| Lachnospiraceae FCS020 | GASTRODU | 12 | 0.87 (0.58-1.30) | 0.510 | 0.83 (0.68-1.01) | 0.060 | 0.85 (0.73-0.99) | 0.040 |
| Lachnospiraceae UCG004 | GASTRODU | 12 | 1.02 (0.51-2.06) | 0.950 | 1.09 (0.87-1.36) | 0.470 | 1.19 (1.00-1.40) | 0.048 |
| Ruminiclostridium 9 | GASTRODU | 8 | 0.69 (0.24-1.99) | 0.510 | 0.75 (0.56-0.99) | 0.039 | 0.77 (0.61-0.96) | 0.019 |
| Catenibacterium | DU | 4 | 2.25 (0.11-46.60) | 0.650 | 1.24 (0.94-1.63) | 0.130 | 1.31 (1.05-1.63) | 0.018 |
| Clostridiumsensustricto 1 | DU | 6 | 0.49 (0.14-1.70) | 0.320 | 0.80 (0.52-1.22) | 0.300 | 0.65 (0.42-1.00) | 0.048 |
| Collinsella | DU | 9 | 0.80 (0.24-2.67) | 0.720 | 0.72 (0.46-1.12) | 0.140 | 0.69 (0.50-0.95) | 0.024 |
| Ruminiclostridium 9 | DU | 8 | 0.66 (0.12-3.57) | 0.650 | 0.65 (0.42-1.01) | 0.050 | 0.68 (0.48-0.97) | 0.031 |
| Parabacteroides | GJU | 5 | 0.16 (3.52e-5-7.51e+02) | 0.700 | 0.22 (0.04-1.28) | 0.090 | 0.22 (0.06-0.84) | 0.027 |
| Bilophila | GJU | 13 | 8.22 (0.14-4.73e+02) | 0.330 | 3.31 (1.10-9.97) | 0.033 | 3.45 (1.52-7.81) | 0.003 |
