# Supplementary material for: Genome-wide Mendelian randomization identifies putatively causal gut microbiota for multiple peptic ulcer diseases
Source: Front Immunol. 2023 Oct 5;14:1260780. doi: 10.3389/fimmu.2023.1260780 (PMC10586326; doi:10.3389/fimmu.2023.1260780)
Supplement: Supplementary file 3 [file Table_2.pdf]

Stable2: Reverse causality between gut microbiota and PUDs

Exposure	Outcome	SNP (n)	IVW	MR Egger	WM
			P-value	P-value	P-value
OESU	Eubacterium hallii	7	0.301	0.953	0.761
OESU	Flavonifractor	7	0.723	0.822	0.924
OESU	Ruminiclostridium 6	7	0.780	0.068	0.765
OESU	Ruminococcaceae UCG013	7	0.334	0.548	0.264
GU	Lachnospiraceae UCG004	15	0.249	0.784	0.519
GASTRODU	Lachnospiraceae FCS020	17	0.886	0.762	0.728
GASTRODU	Lachnospiraceae UCG004	17	0.262	0.597	0.205
GASTRODU	Ruminiclostridium 9	17	0.901	0.897	0.963
DU	Catenibacterium	16	0.867	0.029	0.467
DU	Clostridiumsensustricto 1	16	0.066	0.495	0.202
DU	Collinsella	16	0.580	0.177	0.526
DU	Ruminiclostridium 9	16	0.907	0.385	0.767
GJU	Parabacteroides	3	0.364	0.752	0.443
GJU	Bilophila	3	0.625	0.688	0.558
